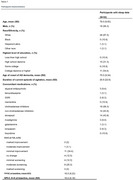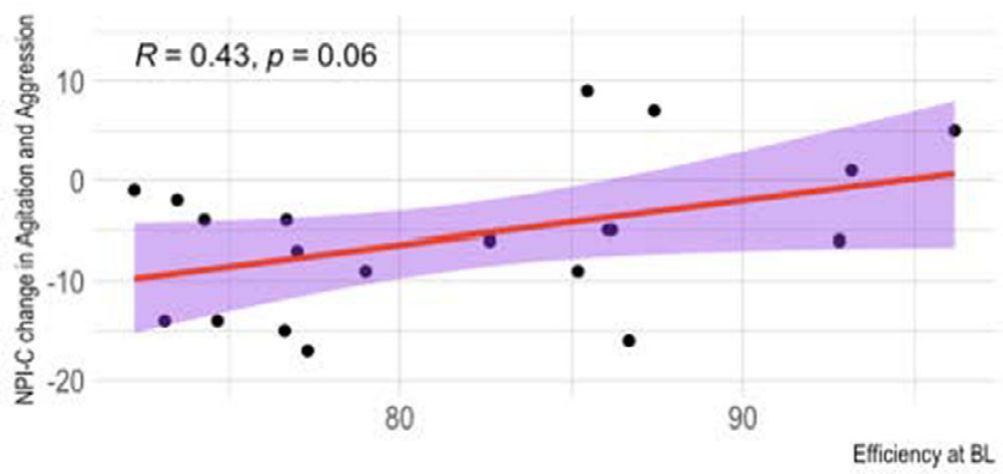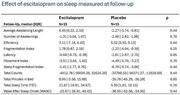# Sleep variables in the S‐CitAD RCT of escitalopram for agitation in Alzheimer's disease

**DOI:** 10.1002/alz70857_106843

**Published:** 2025-12-24

**Authors:** Paul B. Rosenberg, Carol A Manning, Anton P. Porsteinsson, Kostas G. Lyketsos, Sheriza Baksh, Adam P. Spira, Sarah K. Wanigatunga, Amer M. Burhan

**Affiliations:** ^1^ Johns Hopkins University School of Medicine, Baltimore, MD, USA; ^2^ Richman Family Precision Medicine Center of Excellence in Alzheimer's Disease, Johns Hopkins University, Baltimore, MD, USA; ^3^ Department of Psychiatry and Behavioral Sciences, Johns Hopkins University School of Medicine, Baltimore, MD, USA; ^4^ University of Virginia, Charlottesville, VA, USA; ^5^ University of Rochester School of Medicine and Dentistry, Rochester, NY, USA; ^6^ Johns Hopkins University Bloomberg School of Public Health, Baltimore, MD, USA; ^7^ Department of Mental Health, Johns Hopkins Bloomberg School of Public Health, Baltimore, MD, USA; ^8^ Johns Hopkins Bloomberg School of Public Health, Baltimore, MD, USA; ^9^ Toronto Dementia Research Alliance, Toronto, ON, Canada

## Abstract

**Background:**

Sleep commonly is disturbed in Alzheimer's disease (AD) and poor sleep may accelerate AD progression. Disturbed sleep may contribute to neuropsychiatric symptoms (NPS) of AD, including agitation. We examined associations between actigraphic sleep parameters with baseline agitation and changes in agitation with escitalopram treatment among participants in the S‐CitAD trial.

**Method:**

S‐CitAD was a 12‐week randomized controlled trial of escitalopram for agitation in AD. Of 173 S‐CitAD participants, 32 (age = 79 +/‐9.93 years, 43.7 % female, 12.5 % non‐White) had actigraphic sleep data (median 13 nights at baseline). Agitation was measured with the NPI‐C Agitation and Aggression (NPI‐C‐A+A) domains. We measured standard sleep variables (awakening length, number of awakenings, efficiency, fragmentation index, latency, total activity counts during sleep, total minutes in bed, total sleep time, wake after sleep onset).

**Result:**

Of the 32 participants with baseline actigraphy data, 9 had follow‐up at week 12. The actigraphy subset had mean age of 79 +/‐9.9 years, were 56% male, had Mini‐Mental State Exam (MMSE) scores of 18.5 +/‐8.2 and baseline NPI‐C A/A scores of 16.0+/‐6.2 (Table 1). There were no significant associations of sleep variables with baseline agitation or with change in agitation at 12 weeks. Escitalopram responders, defined by a decrease in NPI‐C A/A of ≥ 4 at 12 weeks, had a smaller decline in sleep latency than placebo responders at 12 weeks (*p* = 0.002) Escitalopram participants (responders and nonresponders) had a smaller reduction in sleep latency (0.60 min vs. 2.81) and total minutes in bed (8 min increase vs. 35 min decrease on placebo) relative to people on placebo (Table 2). Although not significant, there was a trend for participants with worse baseline sleep efficiency to have greater 12‐week improvement in agitation. (Figure 1)

**Conclusion:**

We observed no association of sleep variables with baseline agitation or change in agitation with treatment. Escitalopram exposure was associated with modest changes in selected sleep parameters. These findings may inform future studies of agitation in AD as well as targets for future treatments.